# Effects of Contrast Water Therapy on Physiological and Perceptual Recovery Following High-Intensity Interval Swimming in Collegiate Swimmers

**DOI:** 10.3390/sports14010026

**Published:** 2026-01-05

**Authors:** Kazuki Kino, Mitsuo Neya, Yuya Watanabe, Noriyuki Kida

**Affiliations:** 1 Faculty of Sport Study, Biwako Seikei Sport College, Otsu 520-0503, Japan; kino@bss.ac.jp (K.K.);; 2 Faculty of Arts and Sciences, Kyoto Institute of Technology, Kyoto 606-0951, Japan

**Keywords:** contrast water therapy, blood lactate, swimming performance, fatigue recovery, high-intensity interval training

## Abstract

This study examined the effects of contrast water therapy (CWT) on physiological, perceptual, and performance-related recovery in collegiate male swimmers following high-intensity interval training. Fifteen freestyle swimmers (19.3 ± 1.1 years) completed two sessions of five 100 m maximal-effort intervals under two recovery conditions, CWT and passive rest (PAS), in a crossover design. The CWT protocol consisted of 10 alternating immersions in hot (40–41 °C, 60 s) and cold (20–21 °C, 30 s) water. Blood lactate (LA), blood pressure (BP), and subjective fatigue (VAS-FAS) were assessed at multiple time points. Compared with PAS, CWT resulted in significantly lower post-recovery blood LA (7.75 ± 2.08 vs. 10.86 ± 2.86 mmol/L, *p* = 0.002) and reduced subjective fatigue (6.60 ± 1.30 vs. 7.60 ± 0.91 cm, *p* = 0.021), whereas no significant differences were observed in BP or 100-m swimming performance. Individual-level analyses revealed heterogeneous responses, with most swimmers demonstrating improved lactate clearance and reduced fatigue following CWT, although performance responses varied among participants. These findings indicate that CWT facilitates physiological and perceptual recovery without producing immediate performance enhancement. CWT may be considered a practical short-term recovery option for competitive swimmers, although its effectiveness likely depends on individual response characteristics. Further research involving larger and more diverse samples is warranted to clarify optimal application parameters and individual recovery profiles.

## 1. Introduction

Competitive swimming is a sport in which athletes race a designated distance, typically 50 to 1500 m, using specific strokes. The duration of exertion varies considerably by event, ranging from approximately 20 s to more than 15 min. This range requires engagement of multiple physiological energy systems. Sprint events rely predominantly on anaerobic metabolism, whereas long-distance events depend mainly on aerobic metabolism. Regardless of event type, competitive swimmers undergo prolonged, high-intensity training sessions [1,2,3]. Daily training volumes reportedly range from 9000 m to 18,000 m, placing substantial physical demands on athletes [4]. Heated indoor pools also allow year-round competition and training, effectively lengthening the competitive season. These developments underscore the importance of effective recovery strategies to mitigate fatigue and maintain performance [5].

Optimizing recovery strategies is therefore essential for maximizing performance and training efficiency. In recent years, various recovery methods have been developed to meet these growing demands. Among them, water immersion-based recovery techniques have gained particular attention for their potential to reduce post-exercise fatigue [6,7]. However, Parouty et al. [7] reported that prolonged cold-water immersion may reduce the core temperature and impair subsequent performance. Water-immersion methods utilize the physical properties of water (hydrostatic pressure, buoyancy force, and thermal stimulation [hot/cold]). These factors enhance circulation, reduce muscle inflammation, and alleviate perceived fatigue [8,9]. Hydrostatic pressure facilitates venous return and cardiac output, promotes lactate clearance and reduces inflammation [8]. The buoyancy force decreases the gravitational force of the musculoskeletal system, reducing mechanical stress and energy expenditure [9]. Alternating thermal stimuli can modulate skin temperature, relieve muscle soreness and inflammation, and promote relaxation through enhanced blood flow [10,11].

Contrast water therapy (CWT), which alternates between hot and cold water immersion, is widely used to promote blood flow, enhance autonomic regulation, and facilitate rapid recovery [11]. Several studies have shown that CWT reduces muscle soreness, alleviates fatigue, and improves subjective recovery [12,13,14,15,16]. However, the effectiveness of CWT varies depending on the sport, protocol parameters, and individual characteristics, making consistent conclusions difficult. Research focusing specifically on competitive swimmers is limited [4,12]. Although some studies suggest that CWT enhances lactate clearance and reduces fatigue [10,13,14], its direct impact on performance outcomes is still inconclusive.

Swimmers engage in prolonged, sport-specific aquatic training which distinguishes their recovery demands from athletes in other disciplines. Therefore, recovery interventions should be designed from a swimmer-specific perspective. In practice, various recovery methods are used alongside CWT. Icing is often used to reduce localized muscle damage and inflammation. Active recovery is used to enhance circulation and promote metabolite clearance [5]. Massage and compression garments are frequently used to decrease perceived fatigue and muscle soreness [17]. Compared with these methods, CWT uniquely combines hydrostatic pressure with alternating thermal stimulation. This combination may provide simultaneous benefits for circulation enhancement and inflammation control [9]. Thus, CWT represents a practical and potentially effective recovery approach for swimmers.

Therefore, this study aimed to investigate the effects of CWT following high-intensity 100-m training sessions in male collegiate freestyle swimmers. We evaluated its impact from multiple perspectives, including swimming performance, circulatory function, and subjective fatigue index. By standardizing water temperature and immersion protocols, the present study aimed to enhance the practical applicability of CWT and contribute to evidence-based recovery strategies in competitive swimming.

## 2. Methods

### 2.1. Participants

The fifteen male collegiate swimmers (age: 19.3 ± 1.1 years; height: 172.4 ± 4.5 cm; body mass: 68.4 ± 5.9 kg) specializing in 100 m and/or 50 m free style (front crawl stroke) participated in this study. All swimmers were members of teams competing in Japan’s collegiate second division and had an average of 11.1 ± 2.7 years of competitive swimming. Their weekly training hours during this research period were 12 ± 2 h/week. The physical characteristics and personal best times of the participants are presented in Table 1.

Prior to participation, all individuals received verbal and written explanations of the study’s purpose, procedures, and right to withdraw at any time. Written informed consent was obtained from all the participants. This study was approved by the Research Ethics Committee of the Faculty of Physical Education at Tenri University (Approval No. H29-017).

### 2.2. Experimental Timeline

**Figure 1:**
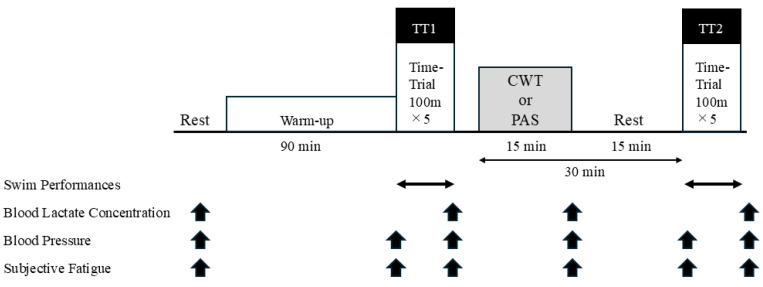
Timeline of experimental protocol. The black arrows indicate the timing for performing each measurement.

The experimental timeline is shown in Figure 1. A crossover design was adopted, with each participant completing both CWT and seated rest (passive rest [PAS]) conditions on separate days. The interval between each participant’s CWT and PAS trials was seven days.

Before the intervention, resting blood pressure (BP), blood lactate concentration (LA), and subjective fatigue index (measured using the Fatigue Assessment Scale [FAS]) were recorded. Participants then completed a 90-min pool-based warm-up. The warm-up duration in this study (≈90 min including dry-land activation and in-water swimming) reflects the standard collegiate swimming practice at the authors’ institution. Prior research on competitive swimmers has also reported warm-up routines ranging from 1000 to 2000 m, often combined with land-based activation before high-intensity sessions, which aligns with the present protocol [1,2].

Following the warm-up, the swimmers performed the first five 100-m front crawl stroke interval swims (work-to-rest ratio of 1:0.5) and were verbally instructed to maintain maximal effort (TT1) throughout. Upon completion of TT1, BP, LA, and FAS were measured, followed by either CWT or PAS

Thirty minutes after the intervention, the second five 100-m interval swims (TT2) were conducted. BP, LA, and FAS were measured again after completion of TT2. All tests were performed in a 25-m indoor pool facility maintained at an ambient temperature of 25–27 °C and relative humidity of 63–68%. The pool temperature maintained between 28.5–29.0 °C.

To ensure that all participants performed the 100-m trials at maximal intensity, swimmers were instructed to swim at an “all-out” pace, and subjective exertion was assessed using the FAS immediately after each bout. In addition, the 100-m performance times recorded in TT1 and TT2 were within the expected range relative to each swimmer’s personal best, indicating appropriate effort and compliance with the protocol. Two adjacent pools (approximately 180 × 120 cm) located in the indoor pool were used for hot (40–41 °C) and cold (20–21 °C) immersion, positioned 1 m apart to ensure transfer times under 5 s. The participants remained seated and fully immersed on their shoulders. Conversation during the immersion was not permitted. The selection of the CWT protocol used in this study was based on established physiological mechanisms associated with thermal contrast. Hot water immersion at approximately 40 °C promotes peripheral vasodilation and increased blood flow, whereas cold immersion at approximately 15 °C induces vasoconstriction and augments central venous return. Alternating these conditions produces rhythmic fluctuations in vascular tone, which may facilitate metabolite clearance and accelerate cardiovascular recovery. Previous studies have reported that protocols with a hot-to-cold duration ratio of approximately 2:1 and ending with cold immersion can optimize post-exercise recovery responses in trained athletes [18,19]. Therefore, the present study employed 10 cycles of 60 s hot and 30 s cold immersion, ending with cold, to reflect commonly used practice in competitive swimming settings. Additionally, the water temperature and immersion duration were determined in accordance with previous findings, suggesting that ending with cold water may be more beneficial [9,20].

### 2.3. Physical Characteristics, Swimming Performance, Circulatory Function, and Subjective Fatigue

Body mass, body fat percentage, and lean body mass were measured using a multi-frequency body composition analyzer (MC-780A, TANITA Corporation, Tokyo, Japan) in the measurement room before the pool protocol.

A 60-fps camera (HDR-CX680, SONY, Tokyo, Japan) was positioned at the center of the indoor pool to record the swimmers’ performances. Swim time (ST), stroke count (SC), swim velocity (SV), and stroke length (SL) were assessed at each 100-m intervals. Manual stopwatches were used to measure the time (sec) taken to swim 100 m (ST). The number of strokes per 100 m was counted visually from the video footage of each 100 m swim (SC strokes). The speed during the 100 m swim (SV m/s = 100 m/ST s) and the distance covered per stroke (SL m/strokes = 100 m/SC strokes) were then calculated. SC was defined as the number of single-arm strokes, and SL was calculated by dividing the swim distance by the total SC.

Blood LA and BP were measured at rest, and pre- and post-intervention. Resting BP was assessed in a seated position using an automatic sphygmomanometer (HEM-720, OMRON, Kyoto, Japan), that recorded systolic (SBP) and diastolic blood pressure (DBP) values.

Blood LA levels were assessed from fingertip capillary blood samples using a portable lactate analyzer (Lactate Pro, LT-1730, ARKRAY, Kyoto, Japan), and measured immediately after BP assessment at rest and 3 min after training.

Subjective fatigue was assessed using a visual analog scale (VAS) within the Fatigue Assessment Scale (FAS) introduced by Vaile, J. [11], which was administered within 10 min after rest and training. The scale consisted of a 100-mm horizontal line with numerical indicators every 10 mm; the right end (100 mm) represented “the most severe fatigue ever experienced.”

### 2.4. Statistical Analysis

All data were expressed as mean ± standard deviation (SD). A two-way analysis of variance (ANOVA) with repeated measures was conducted to examine the effects of condition (CWT vs. PAS) and time (Figure 1) on swimming performances and physiological variables. The Mauchly’s test was used to assess sphericity when violated, Greenhouse-Geisser correction was applied, and adjusted degrees of freedom were used for the F-tests. Effect sizes were calculated using partial eta squared (η*p*^2^). When significant interactions were observed, Bonferroni-adjusted post hoc comparisons were performed for both within- and between-group comparisons. Statistical significance was set at *p* < 0.05. All analyses were performed using IBM SPSS Statistics (version 27, IBM Corp., Armonk, NY, USA).

### 2.5. Individual Response Analysis

To examine inter-individual variability in recovery responses, blood LA and FAS measured at the predefined primary evaluation time point (Intervention Post) were analyzed at the individual level.

For each participant, values under PAS and CWT conditions were plotted to visualize responder–non-responder patterns. For swimming performance, the mean time of the five repetitions during TT2 under each condition was used to evaluate individual performance responses.

These analyses were conducted to illustrate individual adaptations without altering the primary statistical framework of the study.

## 3. Results

### 3.1. Effects on Swimming Performance

**Figure 2:**
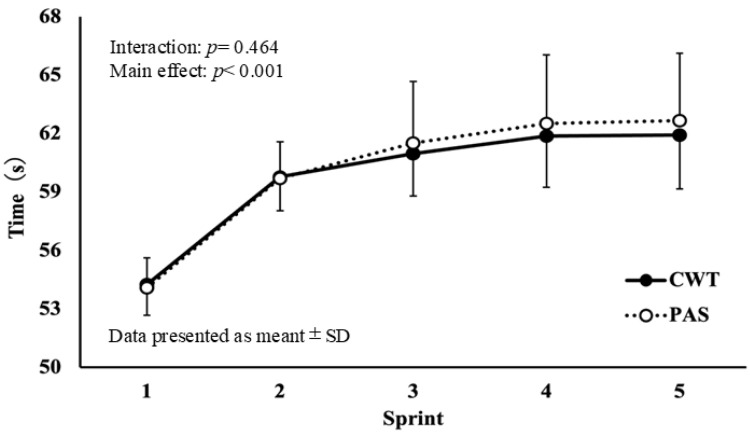
Changes in the swimming time for the 100 m front crawl stroke in TT1.

**Figure 3:**
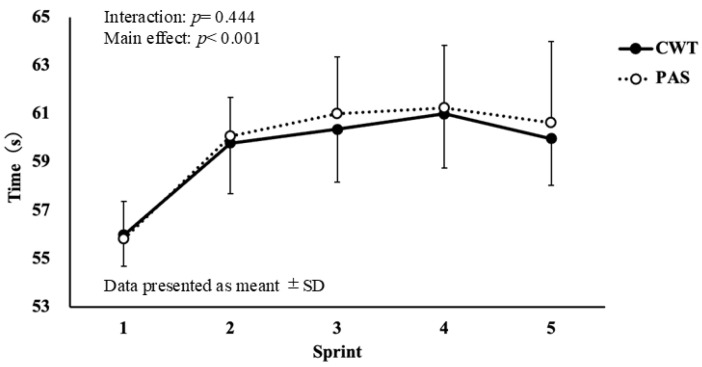
Changes in the swimming time for the 100 m front crawl stroke in TT2.

The ST results for TT1 are shown in Figure 2. No significant interaction was observed between time and condition (F = 1.234, df = 34.857, *p* = 0.464, η*p*^2^ = 0.022). The ST results for TT2 are shown in Figure 3. No significant interaction was observed between time and condition (F = 1.778, df = 49.778, *p* = 0.444, η*p*^2^ = 0.028).

Analysis of SV during TT1 showed no significant interaction was found in TT1 nor TT2 (TT1, F = 1.267, df = 35.489, *p* = 0.487, η*p*^2^ = 0.017; TT2, F = 1.916, df = 53.638, *p* = 0.669, η*p*^2^ = 0.023).

While a significant interaction between time and condition was found (F = 1.806, df = 50.557, *p* < 0.01, η*p*^2^ = 0.201) in SL at TT1, no significant interaction was found at TT2 (F = 2.781, df = 77.857, *p* = 0.349, η*p*^2^ = 0.012).

Regarding SC, no significant interaction was found in TT1 nor TT2 (TT1, F = 2.277, df = 63.749, *p* = 0.484, η*p*^2^ = 0.017, η*p*^2^ = 0.017; TT2, F = 2.240, df = 62.726, *p* = 0.362, η*p*^2^ = 0.032).

### 3.2. Circulatory Function

**Figure 4:**
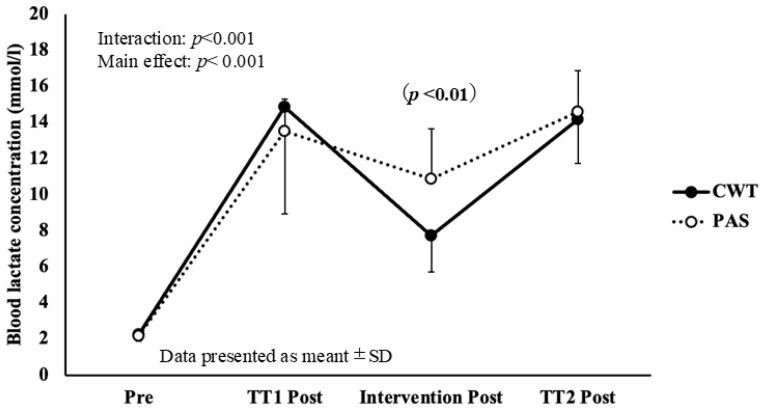
Changes in blood LA across measurement points.

No significant interaction between time and condition for either SBP or DBP was found. Changes in blood LA across measurement points are shown in Figure 4.

A significant interaction was observed for LA (F = 3.84, *p* < 0.001, η*p*^2^ = 0.245), indicating that blood LA after CWT (7.75 ± 2.08 mmol/L) was significantly lower than it was after PAS (10.86 ± 2.86 mmol/L, *p* = 0.002).

### 3.3. Subjective Fatigue

**Figure 5:**
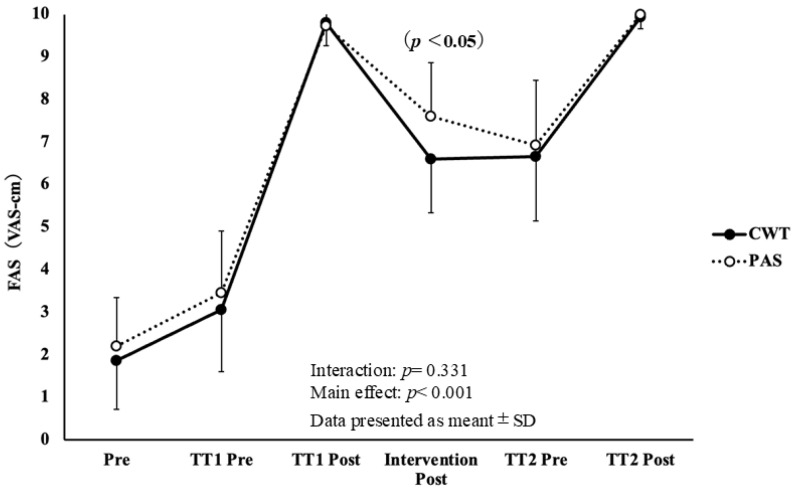
Changes in subject fatigue FAS (VAS-cm) across measurement points.

The effects of the interventions on subjective fatigue assessed using FAS are shown in Figure 5. No significant interaction between time and condition was observed (F = 2.800, df = 78.405, *p* = 0.331, η*p*^2^ = 0.040). However, pairwise comparisons revealed that FAS after CWT (6.60 ± 1.30 VAS-cm) was significantly lower than it was after PAS (7.60 ± 0.91 VAS-cm, *p* = 0.021).

### 3.4. Individual Responses

**Figure 6:**
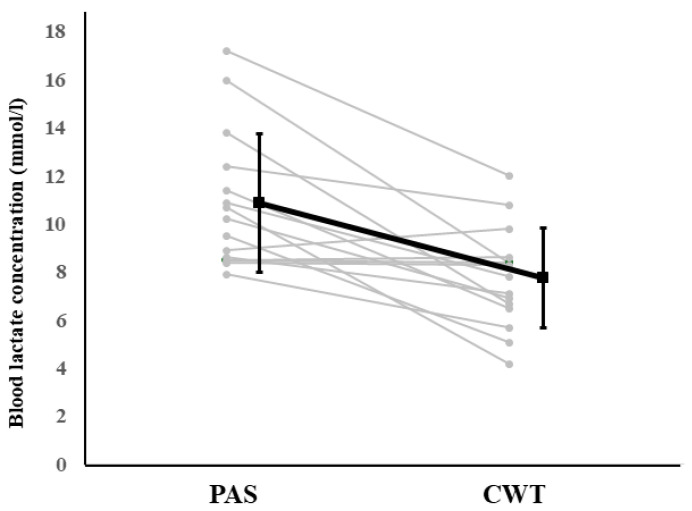
Individual responses in blood LA at Intervention Post between PAS and CWT conditions. Each grey line represents individual participants, and the black line indicates the mean ± SD.

**Figure 7:**
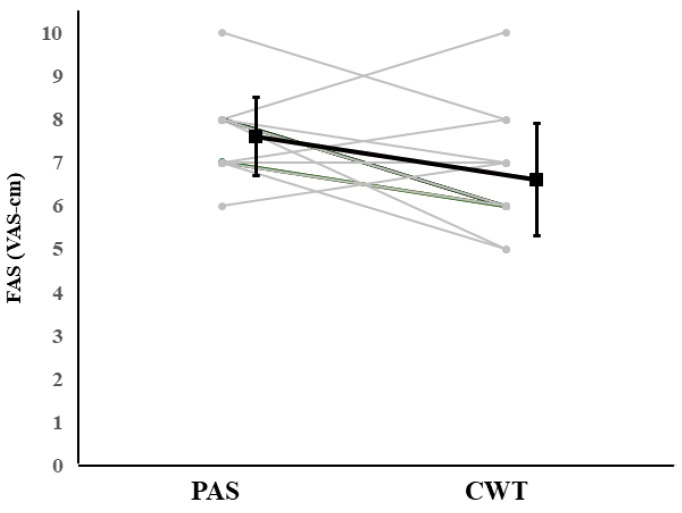
Individual responses in FAS (VAS-cm) at Intervention Post between PAS and CWT conditions. Each grey line represents individual participants, and the black line indicates the mean ± SD.

**Figure 8:**
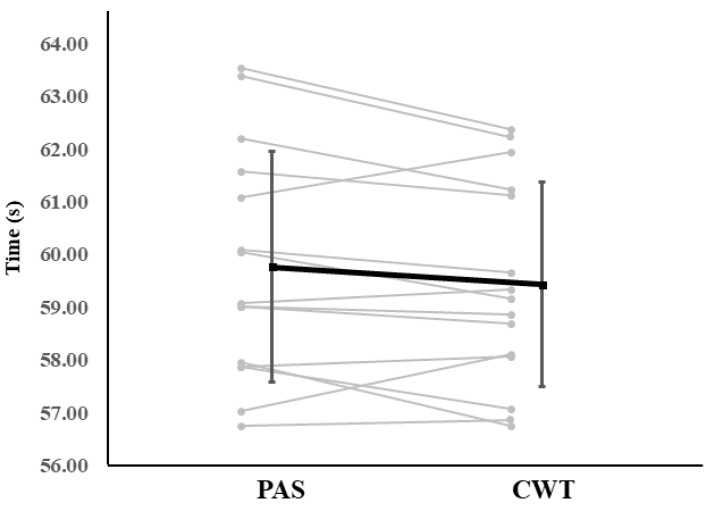
Individual responses in mean 100-m swim time during TT2 between PAS and CWT conditions. Each grey line represents individual participants, and the black line indicates the mean ± SD.

To illustrate inter-individual variability in recovery outcomes, individual values for blood LA, FAS, and mean 100-m swim time during TT2 were compared between PAS and CWT conditions (Figure 6, Figure 7 and Figure 8).

For blood LA at Intervention Post, 13 out of 15 participants showed lower values following CWT compared with PAS, whereas two participants exhibited little change or slightly higher values.

A similar pattern was observed for subjective fatigue, in which 11 participants reported reduced fatigue after CWT, while four showed minimal or increased fatigue levels.

Based on the mean 100-m swim time during TT2, ten swimmers recorded faster times under CWT, whereas five swimmers showed slower times compared with PAS.

These individual data demonstrate that responses differed across participants for physiological (blood lactate), perceptual (fatigue), and performance (swim time) outcomes across the two recovery conditions.

Detailed individual data (mean, standard deviation, and 95% confidence interval values for each variable) are presented in Table A1 and Table A2.

## 4. Discussion

### 4.1. Swimming Performance

This study investigated the effects of CWT on swimming performance, circulatory function, and subjective fatigue index following high-intensity 100-m training swims in collegiate swimmers. Although CWT facilitated blood lactate clearance and reduced subjective fatigue, it did not improve swim performance or blood pressure compared with passive rest.

The relatively long warm-up performed before TT1 may have contributed to pre-fatigue, and influenced early technical variables such as SL. Although this reflects typical competitive swimming practice, accumulated fatigue may have reduced sensitivity to detect performance differences between recovery conditions. Consistent with this, no significant differences were observed in swim time or most stroke variables, except for an interaction in SL during TT1, suggesting that short-term CWT does not immediately enhance performance. These findings align with Versey et al. [19], who reported that acute CWT supports recovery without improving immediate performance.

Three previous studies reported the performance-enhancing effects of water-based recovery interventions [20,21,22]. For example, Zeinab [22] observed improved repeated sprint performance in elite swimmers when CWT was applied between 100-m sprints. Protocol differences may explain discrepancies with the present findings, as earlier studies typically used single maximal sprints relying primarily on ATP–CP and glycolytic systems, while the current protocol used repeated maximal intervals that place greater demands on aerobic energy supply and recovery capacity. These metabolic differences may partly account for the inconsistent performance outcomes across studies.

### 4.2. Physiological Responses

CWT promoted blood lactate clearance, likely through enhanced peripheral blood flow induced by alternating thermal stimulation. High-intensity interval swimming activates glycolytic energy pathways, leading to increased lactate production [20].

Although elevated LA concentrations were traditionally viewed as a primary cause of fatigue [23], more recent studies indicated intramuscular acidosis, rather than lactate itself, plays a greater role in muscle fatigue [24,25]. Nevertheless, a high LA is associated with impaired muscle contractility and reduced exercise performance [26,27]. Aujouannet et al. [28] reported that in elite male swimmers, higher post-exercise blood LA level was associated with a lower stroke index. Elevated blood LA may decrease body fluid pH, inhibit glycolytic ATP production, and impair muscle contraction, thereby delaying physical recovery after exercise [29]. These findings underscore the importance of promoting lactate clearance as an effective post-exercise recovery strategy. Future research should investigate whether enhanced lactate clearance through CWT influences performance over extended recovery periods.

CWT has been reported to increase peripheral blood flow [30] and intramuscular circulation [31]. Although the precise vascular responses to alternating thermal exposure remain unclear, several studies have suggested that repeated vasoconstriction and vasodilation during CWT enhance blood circulation and lactate clearance [11,30,32,33]. Consistent with these findings, we observed significantly lower post-intervention blood LA levels following CWT than after PAS. This finding supports the hypothesis that CWT promotes recovery by facilitating lactate removal through enhanced blood flow.

However, it has also been proposed that increased blood flow during CWT primarily affects superficial tissues, with limited influence on deeper muscle circulation [34]. Therefore, further research is needed to clarify the extent to which CWT affects lactate clearance, particularly in deeper muscle tissues.

Previous studies on post-exercise CWT have reported comparable findings. Rasooli et al. [35] demonstrated that active recovery accelerated lactate clearance in 17 elite swimmers after maximal 200-m freestyle efforts, whereas Mark et al. [36] found that both active recovery and CWT significantly reduced blood LA after a Wingate ergometer test in 14 collegiate hockey players. Similarly Vaile et al. [11] reported that cold-water immersion at 10–15 °C facilitated lactate clearance.

Although the cold-water temperature used in the present study (approximately 20 °C) was slightly higher than that used in previous protocols, 10 cycles of CWT resulted in improved lactate clearance. These results suggest that CWT may achieve recovery effects comparable to active recovery while imposing a lower physical burden than cold-water immersion alone. Consequently, CWT appears to be a practical and effective method for promoting recovery in settings where a turnaround between training sessions is required.

No significant changes were observed in SBP or DBP after CWT or PAS. While thermal stimulation may influence vascular responses [31], it appears that CWT primarily affects peripheral rather than systemic hemodynamics. Few studies have directly evaluated blood pressure responses to contrast water therapy (CWT) in athletes. Mark et al. [36] reported that, following high-intensity exercise, full-body CWT facilitated the rapid return of blood pressure to baseline levels during recovery, which is consistent with the present findings. However, because blood pressure has rarely been assessed as a primary outcome in previous studies, further studies are needed to clarify the hemodynamic effects of CWT.

### 4.3. Subjective Fatigue

This study demonstrated a reduction in perceived fatigue following CWT. Previous research has also reported that CWT significantly improves subjective recovery one hour after fatigue-inducing exercise [14], while the effects fluctuate depending on the assessment time point on a 24–72-h scale [10]. Furthermore, immersion therapy has been shown to support subjective recovery via the restoration of parasympathetic activity (HRV) [37], which is consistent with the findings of this study. From a practical perspective, whole-body immersion baths may reduce fatigue more effectively than showers [8]. Li et al. [38] also reported the effects of CWT on fatigue indices. There were differences in subjects, intervention conditions, exercise load, and evaluation indicators between these two studies and the present study.

Although CWT produced greater reductions in blood lactate concentration and subjective fatigue compared with passive rest, these benefits did not translate into improvements in 100-m performance. This discrepancy can be explained by several factors. First, the 30-min recovery window prior to TT2 may have been insufficient for physiological advantages such as enhanced lactate turnover to meaningfully influence neuromuscular performance. Previous studies have shown that performance restoration following high-intensity swimming often requires longer recovery periods, particularly when central and peripheral fatigue coexist. Second, performance in short maximal swims is strongly determined by anaerobic capacity, neuromuscular power, and technical execution, which may be less sensitive to acute metabolic changes alone. Third, the single significant interaction observed for stroke length during TT1 may reflect subtle technique adjustments or transient fatigue-related compensations that were not large enough to affect overall swim time. Future studies incorporating biomechanical and electromyographic measures may help clarify these mechanisms.

In addition to these group-level findings, examining individual patterns of physiological and perceptual responses provides further insight into how swimmers differ in their recovery profiles.

### 4.4. Individual Responses

The individual response analysis provided additional insight into the variability of physiological, perceptual, and performance outcomes following CWT.

For blood lactate at Intervention Post, thirteen of the fifteen swimmers showed lower values under CWT compared with PAS, whereas two swimmers displayed little or slightly increased values. Similarly, subjective fatigue demonstrated heterogeneous responses, with eleven swimmers reporting reduced fatigue after CWT, while four showed minimal or higher fatigue levels.

Performance responses also varied across participants. Based on mean 100-m swim time during TT2, 10 swimmers recorded faster times under CWT, whereas five swimmers swam slower compared with PAS.

These observations indicate that the effects of CWT were not uniform across individuals, despite the significant group-level reductions in lactate and fatigue.

Such inter-individual variability may reflect differences in thermoregulatory tolerance, peripheral circulation, autonomic responsiveness, recovery capacity, or prior experience with contrast exposure.

The presence of both responders and non-responders across multiple outcome domains suggests that CWT may be more beneficial for some swimmers than others, and highlights the importance of incorporating individualized monitoring when evaluating or implementing recovery strategies.

These heterogeneous responses underscore the importance of interpreting group-averaged effects alongside individual variability, particularly when applying CWT in performance-oriented training environments.

Taken together, these findings clearly demonstrate that recovery interventions that utilize water temperature stimulation, such as warm water baths or alternating hot and cold baths, are effective in alleviating both physiological and subjective fatigue. Potential mechanisms include promotion of venous return and peripheral circulation via hydrostatic pressure; reduction in muscle and joint load via buoyancy force; autonomic nervous system regulation (HRV recovery) via thermal and cold stimuli; and the “pumping effect” from alternating hot and cold exposure. However, systematic reviews have pointed out variability in effects due to inconsistent protocols (e.g., temperature, ratio, total duration, ending temperature, and intervention timing) and differences in comparison groups [12]. Therefore, aligning the timing of evaluation is crucial for interpretation. Overall, CWT may be a practical method for supporting short-term subjective fatigue recovery. Future studies should control variables such as temperature settings, cycle ratios, total duration, ending temperature, and intervention initiation delays. Direct comparisons with other methods, such as active recovery, should be conducted within the same time window. Moreover, when interpreted within the broader context of athlete recovery, the present findings should also be considered alongside alternative evidence-based modalities such as active recovery, neuromuscular electrical stimulation, and thermotherapy, which have demonstrated comparable or superior effects depending on the training context [36]

## 5. Limitations of This Study

This study has several limitations that should be considered when interpreting the findings.

First, the sample size was relatively small, and although we examined the observed effect sizes post hoc, the statistical power was likely insufficient to detect small-to-moderate differences in performance. Second, although individual responder–non-responder patterns were explored descriptively, the small sample size and single-session design limit the ability to establish stable classifications of responders and non-responders. Third, because participants were not blinded to the recovery condition, subjective measures such as fatigue ratings may have been influenced by expectation effects. Fourth, the CWT protocol employed in this study (10 cycles of 60-s hot and 30-s cold immersion) represents only one of many possible configurations; different temperatures, immersion durations, or hot–cold ratios could yield different outcomes. Fifth, generalizability is limited, as all participants were trained collegiate swimmers; the findings may not extend to elite-level athletes, younger or older individuals, or female swimmers. Finally, the ecological validity of the study is restricted because recovery was applied immediately after a controlled training session, whereas recovery timing and procedures vary considerably in real-world training environments. Future studies should include larger and more diverse samples, incorporate blinding where possible, and compare multiple CWT protocols or alternative recovery modalities.

Although individual response patterns were presented, the small sample size limits the ability to determine stable responder–non-responder classifications, and these patterns should be interpreted with caution.

## 6. Practical Applications

The present study found that CWT did not produce immediate effects on swimming performance or BP. Therefore, its direct utility for enhancing performance immediately after competition may be limited.

However, CWT effectively promoted blood lactate clearance and reduced the subjective fatigue index, suggesting its potential value for medium- to long-term fatigue management and recovery support. In practical settings, individual variability and differences in response due to factors such as water temperature and immersion duration should be carefully considered. Accordingly, CWT should be applied flexibly and in conjunction with other recovery methods to optimize its benefits.

A thorough understanding of the unique characteristics of CWT and its appropriate application based on each athlete’s condition, training load, and recovery goals, is essential to maximize its benefits.

Each recovery protocol in this study was performed immediately after a controlled training session, which may not fully reflect real-world training environments where recovery timing varies depending on daily training volume and coaching schedules.

In high-performance sport settings, the relative effectiveness of various recovery modalities is a critical consideration. Recent comparative work has demonstrated that modalities such as active recovery, neuromuscular stimulation, and thermotherapy can influence post-exercise fatigue and metabolic restoration through different physiological mechanisms [39]. When situating the present findings within this broader context, it is important to note that CWT should not be evaluated solely against passive rest. Rather, its practical utility depends on how it compares with other commonly used recovery techniques in competitive swimming. Although CWT improved lactate clearance and subjective fatigue in the current study, future research should incorporate direct comparisons with alternative evidence-based recovery strategies to determine its relative effectiveness in high-performance environments.

Because individual responses varied across physiological, perceptual, and performance measures, practitioners should monitor athlete-specific reactions to CWT and adjust recovery prescriptions accordingly.

## 7. Conclusions

This study investigated the effects of CWT following a high-intensity 100-m interval swim in collegiate swimmers. The analysis was exploratory and aimed to generate preliminary evidence to guide future controlled trials. Although the CWT did not produce immediate improvements in swimming performance or BP, it significantly promoted blood lactate clearance and reduced subjective fatigue.

These findings suggest that CWT is a practical and effective recovery method that may help maintain athletic performance over time by facilitating both physiological and perceptual recovery. Given the modest sample size and the single-session design, the present findings should be interpreted as exploratory. These results provide preliminary insights into the physiological and perceptual responses to contrast water therapy in collegiate swimmers. Further research is warranted to explore its effectiveness under diverse training conditions and among athletes with varying performance levels, with the aim of establishing optimal protocols for its use in competitive swimming.

The presence of both responders and non-responders suggests that the effectiveness of CWT may vary across athletes, emphasizing the need for individualized recovery approaches.

## Figures and Tables

**Table 1:** Physical characteristics of subjects.

Variables	n = 15
Age (years)	19.3 ± 1.1
Height (cm)	172.4 ± 4.52
Body Mass (kg)	68.4 ± 5.9
BMI	23.0 ± 1.4
Body Fat (%)	12.1 ± 2.4
LBM (kg)	57.2 ± 4.0
PB Time (sec)	52.2 ± 1.2
Swimming Career (years)	11.1 ± 2.7

Values are represented as mean ± SD. BMI, body mass index; LBM, lean body mass; PB, personal best time in 100 m front crawl race.

## Data Availability

The raw data supporting the conclusions of this article will be made available by the authors on request.

## Abbreviations

The following abbreviations are used in this manuscript:

CWT	contrast water therapy
PAS	passive rest
TT1	the first five 100 m maximal effort intervals
TT2	the second five 100 m maximal effort intervals
BP	Blood pressure
LA	lactate concentration
FAS	subjective fatigue index
BMI	body mass index
LBM	lean body mass
PB	personal best time in 100m front crawl race
ST	Swim time
SC	stroke count
SV	swim velocity
SL	stroke length
SBP	Systolic Blood Pressure
DBP	diastolic blood pressure

## Appendix A

### Appendix A.1. Swimming Performance and Stroke Parameters Under PAS and CWT Conditions

**Table A1:** Swim time, stroke length, stroke count and swim velocity under PAS and CWT conditions.

Variables	PAS		CWT	
	TT1	TT2	TT1	TT2
Swim Time-1 (s)	54.08 ± 1.60	55.83 ± 1.56	56.00 ± 1.44	54.25 ± 1.77
Swim Time-2 (s)	59.69 ± 1.93	60.08 ± 1.64	59.80 ± 2.16	59.77 ± 1.72
Swim Time-3 (s)	61.51 ± 3.17	61.02 ± 2.37	60.36 ± 2.24	60.97 ± 2.30
Swim Time-4 (s)	62.52 ± 3.52	61.25 ± 2.61	61.01 ± 2.34	61.87 ± 2.70
Swim Time-5 (s)	62.67 ± 3.46	60.63 ± 3.40	59.98 ± 2.25	61.92 ± 2.84
Stroke Length-1 (m/stroke)	2.36 ± 0.10	2.41 ± 0.14	2.49 ± 0.15	2.42 ± 0.12
Stroke Length-2 (m/stroke)	2.45 ± 0.15	2.48 ± 0.15	2.46 ± 0.12	2.46 ± 0.11
Stroke Length-3 (m/stroke)	2.48 ± 0.15	2.49 ± 0.16	2.44 ± 0.12	2.47 ± 0.13
Stroke Length-4 (m/stroke)	2.54 ± 0.19	2.50 ± 0.15	2.47 ± 0.14	2.49 ± 0.12
Stroke Length-5 (m/stroke)	2.52 ± 0.19	2.45 ± 0.15	2.39 ± 0.16	2.44 ± 0.13
Stroke Count-1 (count)	61.40 ± 4.81	61.93 ± 5.02	61.07 ± 4.48	60.27 ± 5.06
Stroke Count-2 (count)	63.73 ± 3.35	62.80 ± 3.45	63.13 ± 3.56	62.53 ± 2.80
Stroke Count-3 (count)	63.47 ± 3.27	62.67 ± 3.75	63.20 ± 3.51	62.33 ± 2.99
Stroke Count-4 (count)	62.33 ± 3.94	62.60 ± 3.60	62.20 ± 2.88	62.47 ± 3.20
Stroke Count-5 (count)	62.13 ± 4.91	63.13 ± 4.63	63.27 ± 3.60	63.60 ± 4.19
Swim Velocity-1 (m/s)	1.85 ± 0.05	1.79 ± 0.05	1.84 ± 0.06	1.78 ± 0.04
Swim Velocity-2 (m/s)	1.67 ± 0.04	1.66 ± 0.04	1.67 ± 0.04	1.67 ± 0.05
Swim Velocity-3 (m/s)	1.62 ± 0.07	1.64 ± 0.06	1.64 ± 0.06	1.65 ± 0.06
Swim Velocity-4 (m/s)	1.60 ± 0.08	1.63 ± 0.06	1.61 ± 0.06	1.63 ± 0.06
Swim Velocity-5 (m/s)	1.59 ± 0.08	1.65 ± 0.08	1.61 ± 0.07	1.66 ± 0.06

Values are represented as mean ± SD.

### Appendix A.2. Physiological and Perceptual Measurements Under PAS and CWT Conditions

**Table A2:** Blood lactate concentration, perceived fatigue, and blood pressure measurements under PAS and CWT conditions.

Variables	PAS	CWT
LA-Pre (mmol/L)	2.18 ± 0.34	2.26 ± 0.38
LA-TT1-post (mmol/L)	13.54 ± 1.80	13.52 ± 2.06
LA-Intervention-post (mmol/L)	10.86 ± 2.86	7.75 ± 2.08
LA-TT2-post (mmol/L)	14.58 ± 2.43	14.18 ± 2.41
FAS-Rest (VAS-cm)	2.20 ± 1.32	1.87 ± 1.41
FAS-TT1-pre (VAS-cm)	3.47 ± 1.36	3.07 ± 1.62
FAS-TT1-post (VAS-cm)	9.73 ± 0.70	9.80 ± 0.56
FAS-Intervention-post (VAS-cm)	7.60 ± 0.91	6.60 ± 1.30
FAS-TT2-pre (VAS-cm)	6.93 ± 1.16	6.67 ± 1.54
FAS-TT2-post (VAS-cm)	10.00 ± 0.00	9.93 ± 0.26
SBP-pre (mmHg)	135.40 ± 13.26	135.20 ± 10.15
SBP-TT1-Pre (mmHg)	142.47 ± 8.80	139.60 ± 14.24
SBP-TT1-Post (mmHg)	156.40 ± 16.42	154.67 ± 12.92
SBP-Intervention-Post (mmHg)	125.93 ± 14.71	127.67 ± 17.37
SBP-TT2-Pre (mmHg)	128.80 ± 9.95	129.13 ± 14.17
SBP-TT2-Post (mmHg)	149.40 ± 14.73	148.93 ± 13.39
DPB-pre (mmHg)	67.47 ± 8.48	66.20 ± 7.74
DPB-TT1-Pre (mmHg)	72.20 ± 8.46	71.67 ± 7.48
DPB-TT1-Post (mmHg)	62.80 ± 9.62	61.40 ± 9.60
DPB-Intervention-Post (mmHg)	59.73 ± 8.15	57.13 ± 6.79
DPB-TT2-Pre (mmHg)	60.60 ± 6.74	58.13 ± 7.68
DPB-TT2-Post (mmHg)	61.87 ± 6.22	61.27 ± 9.80

Values are represented as mean ± SD.
